# Workplace Exposure to Titanium Dioxide Nanopowder Released from a Bag Filter System

**DOI:** 10.1155/2015/524283

**Published:** 2015-06-01

**Authors:** Jun Ho Ji, Jong Bum Kim, Gwangjae Lee, Jung-Hun Noh, Se-Jin Yook, So-Hye Cho, Gwi-Nam Bae

**Affiliations:** ^1^EcoPictures Co. Ltd., Seoul 137-865, Republic of Korea; ^2^Research & Business Foundation, Sungkyunkwan University, Suwon 440-746, Republic of Korea; ^3^Center for Environment, Health, and Welfare Research, Korea Institute of Science and Technology, Seoul 136-791, Republic of Korea; ^4^Green School, Korea University, Seoul 136-713, Republic of Korea; ^5^School of Mechanical Engineering, Hanyang University, Seoul 133-791, Republic of Korea; ^6^Center for Materials Architecture, Korea Institute of Science and Technology, Seoul 136-791, Republic of Korea; ^7^University of Science and Technology, Daejeon 305-303, Republic of Korea

## Abstract

Many researchers who use laboratory-scale synthesis systems to manufacture nanomaterials could be easily exposed to airborne nanomaterials during the research and development stage. This study used various real-time aerosol detectors to investigate the presence of nanoaerosols in a laboratory used to manufacture titanium dioxide (TiO_2_). The TiO_2_ nanopowders were produced via flame synthesis and collected by a bag filter system for subsequent harvesting. Highly concentrated nanopowders were released from the outlet of the bag filter system into the laboratory. The fractional particle collection efficiency of the bag filter system was only 20% at particle diameter of 100 nm, which is much lower than the performance of a high-efficiency particulate air (HEPA) filter. Furthermore, the laboratory hood system was inadequate to fully exhaust the air discharged from the bag filter system. Unbalanced air flow rates between bag filter and laboratory hood systems could result in high exposure to nanopowder in laboratory settings. Finally, we simulated behavior of nanopowders released in the laboratory using computational fluid dynamics (CFD).

## 1. Introduction

It is estimated that millions of new workers and researchers will be exposed to engineered nanomaterials (ENMs) in occupational environments [1]. Various nanoaerosol sources in ENM manufacturing workplaces show complex relations to ENM exposure assessment. While identifying the sources, it becomes necessary to distinguish between ENMs and incidental nanoaerosols [2].

Many recent studies have investigated ENM exposure. Airborne multiwalled carbon nanotubes (MWCNTs) released within a research facility were measured via personal and area air sampling and by real-time aerosol monitoring [3–7]. Lee et al. [8] monitored potential exposure to nanoaerosols at workplaces where titanium dioxide (TiO_2_) and silver (Ag) nanoparticles were manufactured. A series of studies [9–12] attempted to differentiate task- or process-related ENMs from background or incidental nanoaerosols in workplaces.

However, a more urgent problem exists at the research and development stage in laboratories. Many researchers or students who manufacture ENMs using laboratory-scale synthesis systems could be easily exposed to airborne ENMs. Current knowledge indicates that a well-designed exhaust hood system with a high-efficiency particulate air (HEPA) filter can effectively remove ENMs. However, malfunction or failure of this system is not easily detected by regular activity, since ENMs may not be visible with the naked eye even when released at high concentrations. This occurs particularly when an automated ENM harvesting system is nonoperational or the design specification has low collection efficiency of ENMs.

The present study used various real-time aerosol detectors to investigate airborne nanopowders in a laboratory manufacturing TiO_2_. The TiO_2_ nanopowders were produced through a flame synthesis process and collected by a bag filter system for harvesting. We found high possibility of nanopowder exposure in the laboratory environment, due to low particle collection efficiency of the bag filter system. Unbalanced air flow rates were found between the nanopowder harvesting system and the laboratory canopy hood system. Finally, we simulated behavior of nanopowders released in the laboratory using computational fluid dynamics (CFD).

## 2. Materials and Methods

### 2.1. Nanopowder Laboratory

**Figure 1:**
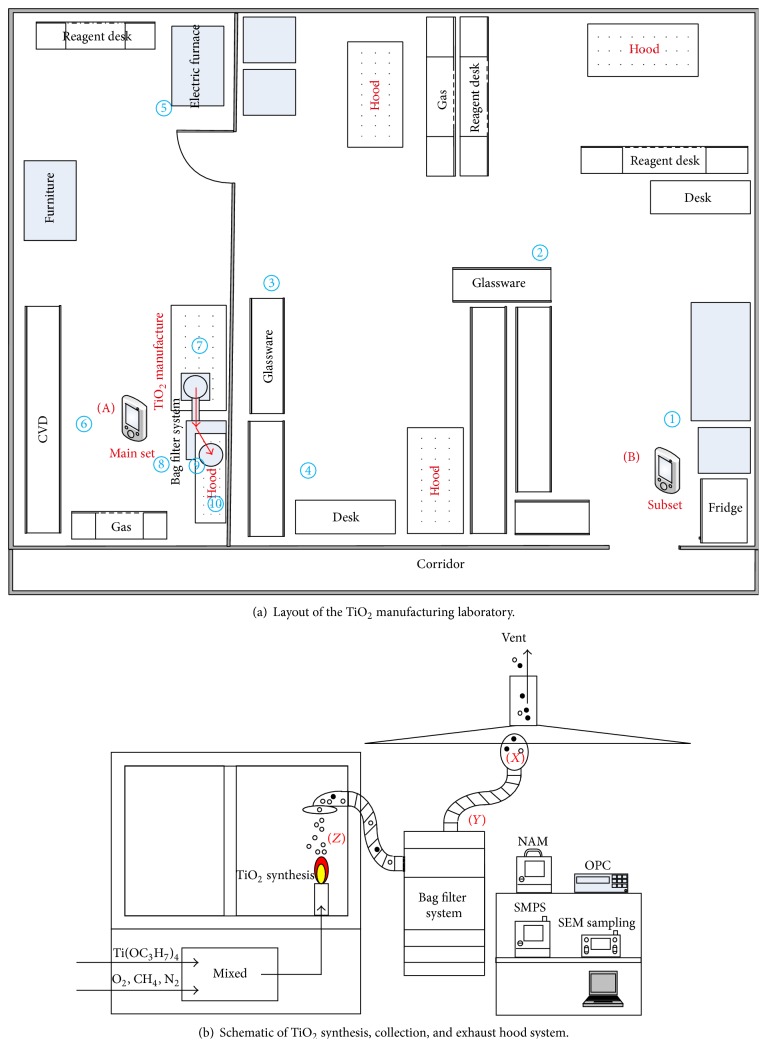
Schematic of TiO_2_ manufacturing laboratory.

The TiO_2_ manufacturing laboratory consisted of a TiO_2_ manufacturing room (3.4 × 9.4 m) and a preparation room (6.85 × 9.4 m). The diameter of the exhaust duct from the canopy hood was 200 mm and flow rate of exhaust air was ~20 m^3^/min. As shown in Figure 1, measurements were carried out at two monitoring positions on September 9-10, 2013. The main position A was near the flame synthesis system; simultaneous monitoring was conducted at subposition B, located near the entrance door of the laboratory, which was connected to a corridor. Comparing the data obtained at the main and subpositions showed whether nanopowders originated from the TiO_2_ manufacturing process. The building had a central air-conditioning system. However, the laboratory had no diffusers for supply air and had only local exhaust hoods connected to the roof fan for exhausting contaminated laboratory air. Therefore, the laboratory air was moved naturally or followed hood-induced air streams.

Flame synthesis was carried out in an open chamber. The precursor vapors, titanium (IV) isopropoxide (TTIP), and flammable gases such as CH_4_ were burnt together in a combustion reactor at ~1,200°C. The detailed TiO_2_ manufacturing procedure was reported by Park et al. [13]. The synthesized TiO_2_ nanopowders were automatically collected by a bag filter system, from which TiO_2_ nanopowders were subsequently harvested. The bag filter system had six cartridges made of polyester and an air-pulse was applied regularly to remove TiO_2_ nanopowders collected on the filters. The filtration area was 11.25 m^2^ and filtration velocity was ~3.2 cm/s.

### 2.2. Experimental

Table 1 lists the real-time aerosol detectors used in this study. A scanning mobility particle sizer (SMPS, Nanoscan, model 3910, TSI, USA) was used to determine particle size distribution within the range 10–420 nm. The device measures particle size distribution for a total scan time of 60 s (45 s scan time, 15 s retrace). An optical particle counter (OPC, portable aerosol spectrometer, model 1.109, Grimm, Germany) was used to monitor particle size distribution within the range 0.25–32 *μ*m for every 60 s. In addition, surface area concentrations of particles deposited in the alveolar regions of the lung were measured using a nanoparticle aerosol monitor (NAM, model AeroTrak 9000, TSI, USA) with PM_1_ cyclone for every 60 s. The air was sampled at flow rates of 0.75, 1.2, and 2.5 L/min for SMPS, OPC, and NAM, respectively.

We measured nanopowders at two monitoring positions (main and subpositions) to differentiate task- or process-related nanopowder exposures from the background or incidental nanoaerosols. Three aerosol detectors were set at the main position and two (OPC and NAM) were set at the subposition. A portable aerosol sensor (Discmini, Matter Aerosol, Switzerland) was used to check the instant episode and spatial particle distribution at locations ① to ⑩ in the laboratory. We moved the instrument between locations so that these measurements were not only spatially segregated but also temporally separated. For episodes of increasing concentration, the portable aerosol detector was very helpful in identifying the source locations. The morphology of airborne nanopowders was observed by scanning electron microscopy (SEM, model NOVA 600, FEI with an accelerating voltage of 30 kV). Membrane filter (Isopore membrane filter, pore size of 100 nm) sets were used in air sampling for the SEM analysis, using a personal sampling pump (model GilAir Plus, Sensidyne, LP, USA) at 0.5 L/min.

## 3. Results and Discussion

### 3.1. Task-Based Exposure Characteristics in the TiO_2_ Laboratory

**Figure 2:**
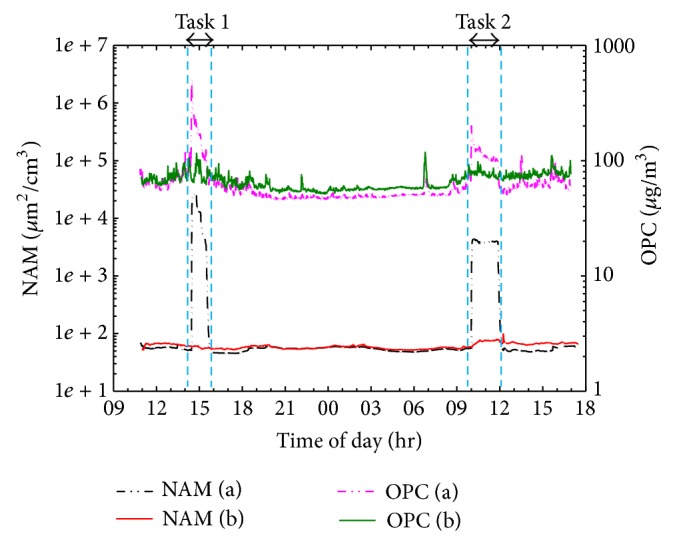
Concentration changes in particle mass from OPC and surface area from NAM during two TiO_2_ synthesis processes.

**Figure 3:**
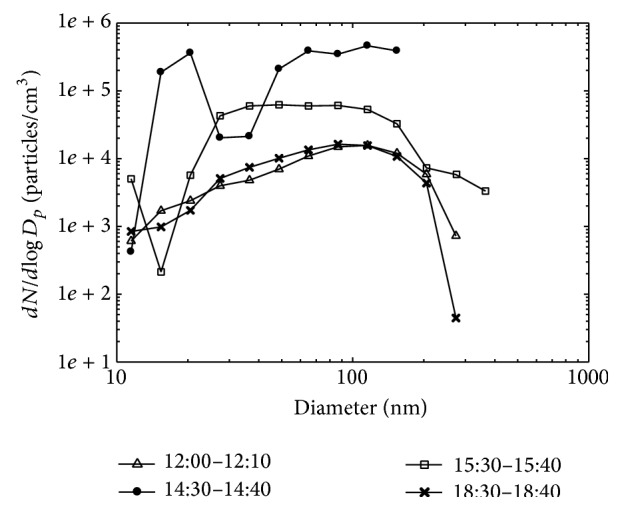
Particle size distributions in the laboratory before, during, and after TiO_2_ synthesis.

Figure 2 shows variations in particle concentrations measured using two real-time aerosol detectors, including the flame synthesis process of tasks 1 and 2. Surface area and mass concentrations of particles showed rapid increase at main position A during TiO_2_ synthesis. In contrast, particle concentrations at subposition B were unchanged. Since synthesized or agglomerated TiO_2_ powders were mostly < 100 nm, as shown in Figure 3, surface area concentration monitored by NAM was more sensitive than PM_10_ mass concentration measured by OPC.

Figure 3 shows the particle size distributions measured using an SMPS. Before and after the synthesis process, the geometric mean particle diameter was approximately 100 nm and the total number concentration was approximately 10,000 particles/cm^3^. However, during the synthesis process, the high number concentration at main position A prevented correct measurement of the particle size distribution. This suggests that the particle number concentration in the laboratory exceeded 10^6^ particles/cm^3^, which is the upper limit of the Nanoscan device. For qualitative information, snap shots of particle size distribution during the synthesis are plotted in Figure 3. At 14:30–14:40, bimodal size distribution indicates that fresh synthesized TiO_2_ nanopowders of ~20 nm and agglomerated TiO_2_ nanopowders of 50–200 nm were released. One hour later, the particle concentration decreased overall and a broad size band appeared.

**Figure 4:**
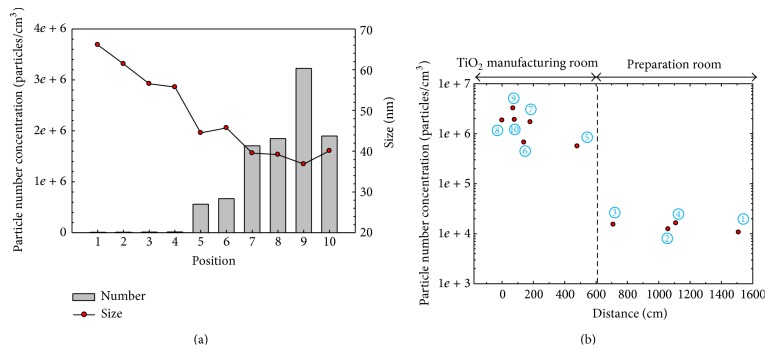
Particle size characteristics, measured by a Discmini, during the synthesis process: (a) particle number concentration and geometric mean diameter, and (b) particle number concentration with distance from the main position A.

Figure 4(a) shows the number concentration and geometric mean diameter of particles, measured using a Discmini, during the second synthesis task (task 2) at the ten locations shown in Figure 1. The geometric mean diameter was approximately 40 nm at the synthesis area, and these manufactured TiO_2_ nanopowders were released to the interior of the laboratory. This means that most of the airborne nanopowders in the synthesis room originated from leakage of the bag filter system during TiO_2_ synthesis. The mean particle diameter was similar to that of TiO_2_ produced by flame synthesis, which was mostly smaller than 50 nm [13]. On the other hand, indoor air in the preparation room showed relatively low particle concentration regardless of the distance from the source location. Here, the particle number concentration and the geometric mean diameter were approximately 10,000 particles/cm^3^ and 60–70 nm, respectively. These data are very similar to those of Figure 3 for the synthesis room prior to the synthesis task. A closed door separated the preparation room from the manufacturing room, with the result that particle number concentration was much higher in the manufacturing room, as shown in Figure 4(b).

Table 2 compares particle concentrations measured at positions A and B using three real-time aerosol detectors. During the task, the total number concentration of particles smaller than 700 nm, measured by a Discmini, near the synthesis system was 200 times higher than that in the preparation room. However, the number concentration of particles larger than 250 nm, measured by an OPC, near the synthesis system was only 1.98 times higher, implying that most nanopowders were smaller than 250 nm.

### 3.2. Evaluation of Bag Filter Performance for Harvesting TiO_2_ Nanopowder

During the synthesis process, we could not evaluate the fractional particle collection efficiency of the bag filter system, due to the high number concentration and high temperature at the inlet of the collection equipment. As shown in Figure 1(b), we measured particle size distribution at both the inlet and outlet of the bag filter system. The performance was evaluated by comparison with indoor aerosol concentrations in conditions without synthesis process. The particle size distribution of indoor aerosols was similar to that shown in Figure 3. The fractional particle collection efficiency, *η*_*f*_, was calculated by

(1) $$\begin{array}{c} \eta_{f}=1-\frac{C_{\text{out}}}{C_{\text{in}}}, \end{array}$$

where *C*_in_ is the number concentration at the system inlet and *C*_out_ is that at the system outlet. We measured inlet aerosols at *Z* for *C*_in_. The aerosols at a position 20 cm inside flexible duct outlet of the system were measured, using a Nanoscan at *X* for *C*_out_.

**Figure 5:**
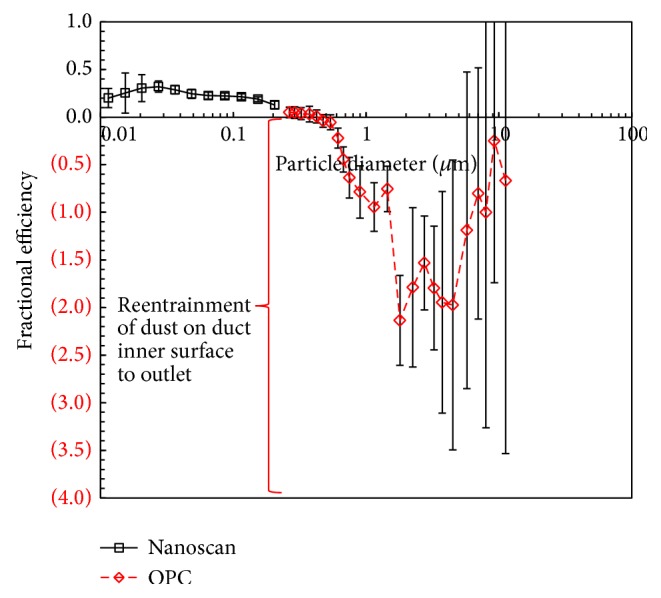
Fractional particle collection efficiency of the bag filter system.

Ten measurements were made sequentially at both the inlet and outlet using SMPS and OPC.  Figure 5 shows the fractional particle collection efficiency of the TiO_2_ collection equipment. Particle collection efficiency at 100 nm was approximately 20%, meaning that much of the TiO_2_ nanopowder generated by the flame synthesis was not collected in the bag filter equipment. For particles larger than 200 nm, the fractional particle collection efficiency of the bag filter system appears to be negative. This finding might be a result of the reentrainment of aggregate particles composed of mainly TiO_2_ nanopowders. Due to the low collection efficiency of the bag filter system, TiO_2_ nanopowders exiting the system were partially deposited on the inner surface of the flexible duct. Subsequently, aggregate particles between 1 and 10 *μ*m were detached from the duct surface and blown into the indoor air during operation of the bag filter system.

**Figure 6:**
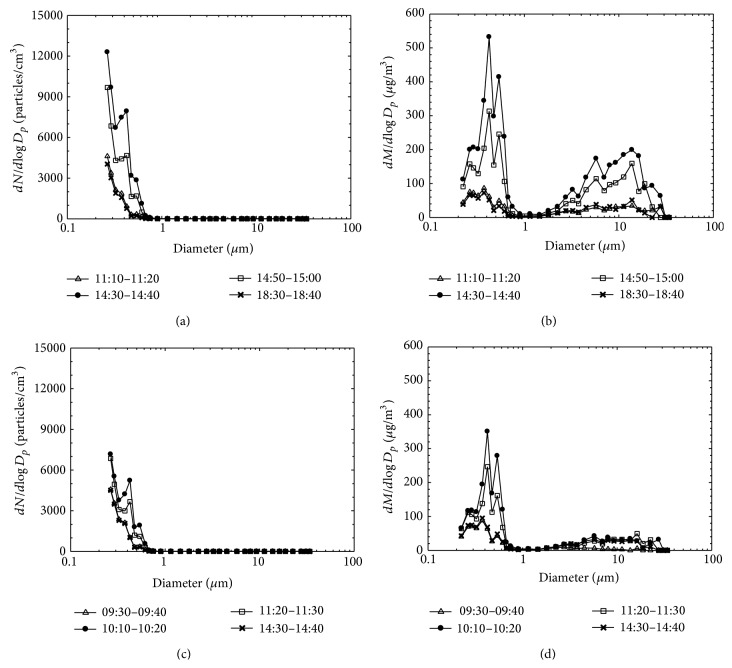
Analysis of particle size distribution measured using the OPC: comparison of number-based particle size distribution (a and c) and mass-based particle size distribution (b and d). Panels (a) and (b) refer to the period including task 1, and (c) and (d) to the period including task 2.

Figures 6(a) to 6(d) show the particle size distributions measured at main position (A) using the OPC during the two synthesis processes in order to analyze reentrainment of TiO_2_ aggregates. As shown in Figures 6(a) and 6(c), significant increase in the number concentration of particles smaller than 0.5 *μ*m was observed for the periods including tasks 1 and 2, respectively. Figures 6(b) and 6(d) show mass-based particle size distribution converted from the data of Figures 6(a) and 6(c), respectively. Here, the mass size distribution was obtained by means of Control Grimm-spectrometer software (v2.5.4). Significant increase in mass concentration of particles between 1 and 30 *μ*m is also seen, providing evidence of reentrainment from the inner surface of the outlet duct in the bag filter system. Due to the low performance of the bag filter system, penetrated TiO_2_ nanopowders were deposited on the inner surface of the outlet duct during the synthesis process. This phenomenon was confirmed by visual inspection. These deposited aggregated TiO_2_ powders might subsequently be reentrained from the outlet duct of the collection system when the system is turned on.

### 3.3. Effect of Unbalanced Air Flow Rates between Nanopowder Harvesting System and Local Hood System

We measured the air flow rate of the bag filter system used for nanopowder harvesting and that of the local hood system described in Figure 1. Air velocity was measured using a multichannel anemomaster (model 1560, Kanomax, Japan) with an omnidirectional probe (model 0964-01/02). The air flow rates were estimated from the three-point velocity data measured at the cross sections of each duct inlet. The air flow rate of the bag filter system was approximately 30 m^3^/min, compared with only 19.2 m^3^/min for the hood system. The TiO_2_ nanopowders that penetrated through the bag filter system were not fully removed by the local hood system; instead, large amounts of TiO_2_ nanopowders were transferred and diffused into the interior of the laboratory, as shown in Figure 2.

### 3.4. Numerical Simulation for Nanopowder Release in the Laboratory

**Figure 7:**
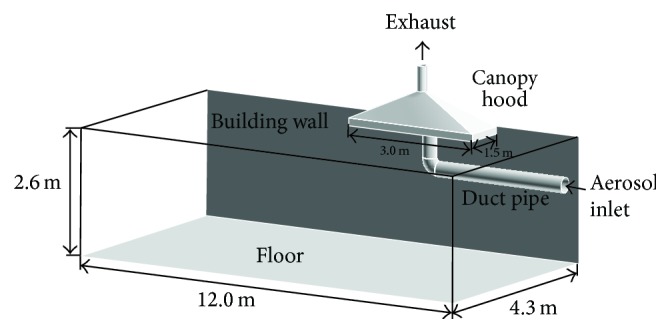
Schematic of calculation domain.

In order to check whether or not aerosols could be effectively exhausted through the canopy hood, aerosol flow near the canopy hood was simulated using FLUENT commercial computational fluid dynamics (CFD) software.  Figure 7 shows the calculation domain. Aerosol was released from a duct pipe with 90-degree elbow, into the space beneath the canopy hood. The duct pipe and canopy hood were placed very close to a side wall of the laboratory room. The inner diameter of the duct pipe was 300 mm, and that of the exhaust duct was 200 mm. In reference to the real situation, the centerline of the upright part of the duct pipe was 200 mm off-center to that of the canopy hood. The flow rate of aerosol released from the duct pipe was 30 m^3^/min, whereas that drawn into the canopy hood was 19.2 m^3^/min. Ambient temperature and pressure were set at 293 K and 101.3 kPa, respectively. The flow was assumed to be three-dimensional, steady, incompressible, and turbulent. The standard k-*ε* turbulence model was employed. The boundary conditions were the velocity inlet condition at the aerosol inlet of the duct pipe, the velocity outlet condition at the exhaust of the canopy hood, and the no-slip condition on the walls of the duct pipe, canopy hood, ceiling, and floor. The no-slip condition was also applied to the laboratory side wall near the canopy hood (dark-gray-colored wall in Figure 7), whereas the symmetry condition was imposed on the other side walls of the laboratory. The convergence criterion for iteratively solving the continuity, momentum, and energy equations was set at 10^−6^. The coupled set of governing equations was iteratively solved by using the finite volume method with SIMPLE algorithm. From the result of the grid dependence test, the number of grids was determined as approximately 7.8 million. After the flow field was obtained, particle trajectories were calculated using the discrete phase models (DPM), based on a Lagrangian reference frame. Particle sizes were selected as 20 nm, 100 nm, and 5 *μ*m. The forces considered to act on the particles were the gravitational force, Brownian force, and Stokes drag force with slip correction.

**Figure 8:**
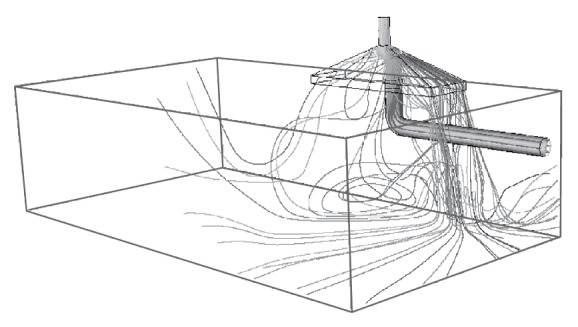
Predicted trajectories of particles injected from the duct pipe inlet.

The fraction of particles exhausted through the canopy hood, the fraction entrained in the room air, and the fraction deposited in the duct pipe are almost same for the three particle sizes. As an example, the results for 5 *μ*m particle size are discussed.  Figure 8 shows the predicted trajectories of 5 *μ*m particles. After exiting the duct pipe, most of the aerosol particles were exhausted through the canopy hood. Some of the particles, however, were introduced to the laboratory room. Looking at the simulation results for the tested particle sizes, only 52% of aerosol particles injected from the duct pipe inlet were estimated to be exhausted through the canopy hood, and about 19% were predicted to be entrained in the air of the laboratory. This was mainly because the flow rate through the canopy hood (19.2 m^3^/min) was less than that through the duct pipe (30 m^3^/min). Therefore, it is of great importance to ensure sufficient suction flow rate of a canopy hood. Meanwhile, approximately 29% of aerosol particles injected from the duct pipe inlet were estimated to be deposited on the inner wall of the duct pipe and the surface of the canopy hood. Particles could be lost in a duct pipe, due to turbulent deposition, sedimentation, inertial deposition, diffusion, and so forth. In addition, particle loss could be exacerbated in an elbow junction, due to the development of the secondary flow. Additionally, some of the particles exiting the outlet of the duct pipe were estimated to be deposited on the canopy hood. Because piling of particles on a surface in high airflow may result in the problem of particle reentrainment, the duct pipe and canopy hood need to be cleaned or replaced periodically.

### 3.5. Source Analysis from SEM Images

**Figure 9:**
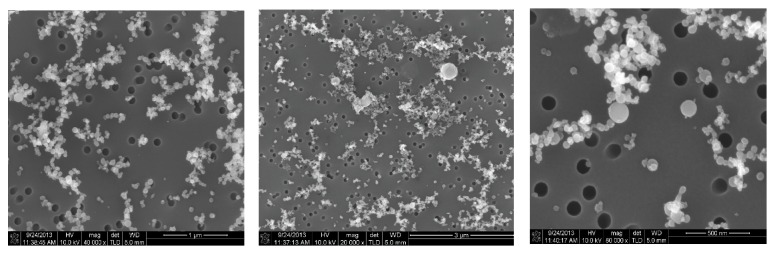
SEM images of powders sampled in the TiO_2_ manufacturing laboratory.

Figure 9 shows SEM images of nanopowders sampled from laboratory air via the Isopore filters (pore size 100 nm). Most particles were spherical TiO_2_ [13]. The diameters of the primary particles ranged from 20 to 150 nm. From Figure 4(a), the geometric mean diameter in the laboratory was approximately 40 nm with polydisperse size distribution. From the SEM image, it was also possible to estimate that many particles were suspended in the air as a form of aggregate, partially by coagulation in the flame zone during the synthesis process and also by reentrainment of aggregate dusts detached from the outlet of the flexible wall-duct.

## 4. Conclusions

In this study, we investigated nanopowder exposure in a laboratory that uses flame synthesis to manufacture TiO_2_. The TiO_2_ nanopowders were collected by a bag filter system for subsequent harvesting. During the manufacturing process, we found high concentrations of nanopowders within the laboratory as a result of low collection efficiency of the bag filter, exacerbated by the lower flow rate of the receiving extraction hood. This was confirmed by CFD simulation, which predicted that large amounts of nanopowders would be released from the bag filter system and be recirculated within the laboratory.

It is noted that the type of equipment used for harvesting nanomaterials is very important in avoiding nanomaterial exposure in manufacturing facilities or workplaces. The performance of harvesting equipment should exceed HEPA grade. In addition, the local hood system should be of appropriate specification and sufficient capacity to fully exhaust the air flow discharged from the harvesting equipment.

## Figures and Tables

**Table 1:** Aerosol detectors used in this study.

	Specifications of instrument	Main set	Subset	Portable aerosol detector
Particle size distribution	10–420 nm	SMPS (Nanoscan, TSI 3910)		
	<10^6^ particles/cm^3^			
	0.25–32 *μ*m	OPC (portable aerosol spectrometer, Grimm 1.109)	OPC (portable aerosol spectrometer, Grimm 1.109)	
	<2 × 10^6^ particles/L			

Particle number concentration	10–700 nm			Discmini (Matter Aerosol)
	<10^6^ particles/cm^3^ @20 nm			
	<5 × 10^5^ particles/cm^3^ @100 nm			

Lung-deposited surface area	10–1,000 nm	NAM (TSI AeroTrak 9000)	NAM (TSI AeroTrak 9000)	
	<10^4^ *μ*m^2^/cm^3^ for A mode			

SEM		Filter sampler (pore size 100 nm)		

**Table 2:** Particle concentrations measured during TiO_2_ synthesis.

	Main set (*A*)	Subset (*B*)	Ratio (*A*/*B*)
Background			
OPC (particles/cm^3^)	717.3 ± 14.0	828.5 ± 15.2	0.87
OPC (*μ*g/m^3^)	49.6 ± 1.1	57.6 ± 1.1	0.86
NAM (*μ*m^2^/cm^3^)	50.1 ± 2.4	53.4 ± 2.0	0.94
Discmini (particles/cm^3^)	—	—	—
Flame synthesis task			
OPC (particles/cm^3^)	1,332.6 ± 188.1	918.2 ± 13.9	1.50
OPC (*μ*g/m^3^)	116.4 ± 18.4	77.3 ± 5.2	1.45
NAM (*μ*m^2^/cm^3^)	3,930.2 ± 243.2	72.3 ± 5.0	54.3
Discmini (particles/cm^3^)	2 × 10^6^	10^4^	200
